# Dependency and Care: Perspectives from the Point of View of Professionals Assessing Situations of Dependency in Spain

**DOI:** 10.3390/ijerph17041178

**Published:** 2020-02-13

**Authors:** Pablo A. Cantero-Garlito, Juan Antonio Flores-Martos, Pedro Moruno-Miralles

**Affiliations:** 1 Faculty of Health Sciences, University of Castilla - La Mancha, 45600 Talavera de la Reina, Spain; Pedro.Moruno@uclm.es; 2 Department of Philosophy, Anthropology, Sociology and Aesthetics, University of Castilla-La Mancha, 45600 Talavera de la Reina, Spain; juanantonio.flores@uclm.es

**Keywords:** long-term care, socio-economic factors, dependency, dependency assessment

## Abstract

Objective: To describe how the assessors of dependency have perceived the process of implementation of the Dependency Act in Spain. Methods: A qualitative method was used to analyse interview data (discourse analysis). Purposive sampling was applied. Sixteen occupational therapists were included, who served as dependency assessors in Extremadura, a region of southern Spain. Data were collected through semi-structured interviews between February and March 2019, focused on the characteristic of the Dependency Act. A discourse analysis of the narrative information was performed using processes of open, axial, and selective coding, as well as the constant comparative method. Results: Three topics were identified: (1) Benefits of implementation, connected with the consideration as subjects of rights and the increase in resources. (2) Difficulties linked to the bureaucratization of the process, the lack of sensitivity of the scale of assessment, and the unequal access to benefits and services. (3) The impact of the 2012 budget cuts on financing and on dependent people and their families. Conclusions: The Dependency Act has established itself as a political tool that has generated important social and economic benefits. However, significant difficulties have emerged, which should be addressed to ensure better care for dependent persons.

## 1. Introduction

The progressive increase in dependent populations in Spain and the resulting need for care have led institutions to develop social, health, and economic policies to prevent, limit, and address the effects of this growth [1]. The care provided to these people represents a major challenge due to both the number of people in need of care and to the economic effort that it implies [2,3]. In December 2008, there were 442,509 people in Spain recognized as dependent. This number increased to 1,385,037 in December 2019, and 72.58% are 65 years of age or older. More specifically, 18.33% are between 65 and 79 years of age, and 54.24% are 80 or older. Further, 65% are women. Of these, it is significant that those over 80 years of age represent 63.92%. On the other hand, 66,618 people under the age of 18 are currently considered as dependent persons (representing 5.92% of the total).

Moreover, the process of implementation of these policies cannot be isolated from the so-called “care crisis” [4], produced by the transformation of the traditional structures on which care was based and which were built on the role given to women at home, in a moment when there is a need for overall reflection of the sustainability of the public system of social and economic benefits given that its cost will amount to 11,339 million euros by 2020, according to the projections of the Commission for the Analysis of the Situation of the Dependency System [5].

Additionally, it has become evident over time that there has been a gradual change of mind about who is responsible for providing care for the people in the situation of dependency. It has been assumed that this care must no longer be provided by the family only, but that it requires active involvement of the State, which has led to the creation of public care policies [6]. It is in this context that Act 39/2006 of the Promotion of Personal Autonomy and Care of Dependent Persons (Dependency Act, from hereon) was adopted on 14 December. This is a legislative package inspired by long-term care policies of European Nordic countries [7] which has becomes the fourth pillar of the Spanish Welfare State, together with the health system, the education system, and the pension system. Its main goal is “equality in the exercise of the subjective right of citizens to the promotion of personal autonomy and care of dependent persons”. Therefore, this Act implies the recognition of a citizen right thanks to which dependent persons are guaranteed access to adequate resources to meet their care needs in an appropriate way. The Act defines dependency as “the permanent state in which people find themselves, for reasons derived from age, illness or disability and linked to the lack or loss of physical, mental, intellectual or sensorial autonomy, thus requiring the care of another person or other persons or significant assistance in performing the basic activities of daily living (ADL, from hereon), or, in the case of people with intellectual disabilities or mental illnesses, other support for their personal autonomy”.

However, as some studies point out [8], the Dependency Act has proved “insufficient in practice both for reasons of design and for funding problems, which have revealed its limitations given the wide variety and case-mix of chronic diseases, excluding a considerable number of people with chronic diseases who cannot receive the benefits provided by the Act”. Likewise, the emergence of the economic crisis, whose impact on the health and inequality of the Spanish population has been remarkable [9], prompted the implementation of public expenditure control measures. These include the Royal Decree/Law 20/2012 (RDL 20/2012, from hereon), which brought about changes in social and health protection structures, and which, as regards our case, constituted a “comprehensive reform” or a “refounding” of the Dependency Act [10]. As of 31 December 2018, 19.2% of dependents were not receiving any benefits or services from the dependency care system, even though they are fully entitled to them. This affects 250,000 people and their families. On the other hand, 30,400 people died without being able to exercise their rights as dependent persons. This means that every day more than 80 dependent persons die without having received any benefits or services.

Few studies have provided in-depth reflection of the process of implementation of the Act from the perspective of the professionals responsible for the application of the Dependency Assessment Scale (DAS) (Baremo de Valoración de Dependencia, BVD, in Spanish), who are the direct agents in the users’ access to the system, regarding the conceptualization of dependency and autonomy underlying the scale of assessment [11,12] used by these professionals [13], as well as the impact on its management process [4] of the different legislative changes made during the years when the economic crisis in Spain prompted restrictive political measures on public expenditure. This Scale has been administered by occupational therapists in many Spanish regions, so they have a first-hand view of both the reality of dependent people and of the different modifications to the Act. In addition, occupational therapists are specialists in the assessment of ADL due to their training. Thereby, they are thus best able to ensure an accurate analysis of the DAS. In this way, those who require help to carry out various ADL will be considered to be in a situation of dependency. Depending on the level of help required, three different degrees of dependency are considered: moderate, severe or high dependency.

As far as we know, this is the first qualitative research that explores how the occupational therapists in charge of assessing the situations of dependency in Extremadura have perceived the implementation of the Dependency Act. No references were found in scientific papers. Considering the impact of the economic crisis on the Spanish population and the mandatory restructuring of the social protection systems, this study, ground-breaking in Europe, may be particularly relevant for understanding similar phenomena in other European countries and for establishing policy measures which provide better guarantees for dependent populations. Therefore, we aimed to explore, on the one hand, how the occupational therapists experienced the development of the Dependency Act, and on the other hand, the effects of austerity policies on the development of the Act and on people receiving care and their families. 

## 2. Methods

### 2.1. Study Design

In order to achieve the objectives of this study, a methodological qualitative approach based on discourse analysis was used [14,15], as well as a phenomenological design [16], which allowed us to obtain detailed information on a topic that had not been sufficiently investigated before. A phenomenological design focuses on the commonality of a lived experience within a particular group [17]. Therefore, it allows describing and enlightening how people understand and comprehend certain phenomena. The primary goal of this design is to obtain an accurate description of the nature of a phenomenon. Therefore, this methodological approach is consistent with the main goals of our study. Thereby, we were able to explore, describe, and understand the meaning that participants gave to their reality and experiences. We conducted theoretical and purposeful sampling, selecting occupational therapists assessing situations of dependency in Extremadura who could provide relevant information on the subject of the study [18]. 

### 2.2. Sample and Data Collection

The Dependency Act entrusted autonomous regions with the task of determining the professional profiles of the technicians responsible for the assessment of dependency situations. In the case of Extremadura, a region in south-western Spain composed of two different provinces (Cáceres and Badajoz), one of the key elements of this task is the occupational therapists who form the 20 assessment teams throughout the region. They apply the DAS to determine people’s capacity to carry out basic ADL in their usual contexts. In this study, we included occupational therapists who work currently or worked in the past as dependency assessors. The selection of participants was carried out following two fundamental criteria: the existence of a large and equitable representation of participants from the different geographical areas of the region (rural-urban, small town-large town), and, on the other hand, to have as many professionals working from the beginning of the Dependency Act implementation in Spain as possible. This allowed for a privileged position with a wide cross-sectional overview of territorial differences and of changes in the dependent population and their families over time. We excluded other professionals who had carried out this work occasionally or for the requirements of the service. This selection was made among assessors from the different health districts of the region. Finally, 16 participants were included: 11 women and 5 men, of which 9 worked in Cáceres, 4 in Badajoz, and 3 no longer worked as assessors (Table 1). All participants assess between 16–18 people per week. Most of them have been in this job since the beginning of the implementation of the Dependency Act.

Data were collected through semi-structured interviews [18], conducted in person, between February and March 2019. We used a script to structure the interviews (Table 2). This outline was open-ended so as to not influence the participants’ responses. Likewise, field notes were collected from the interviewer that included data on the place, as well as additional information provided by the participants before or after the interview.

The initial question invited the participants to talk about the Dependency Act’s contribution to the region. Follow-up questions focused on topics such as the use of the assessment scale, the effects of austerity policies on their work as assessors or their perception of the expansion of dependency care resources.

Using an iterative process, the data collection and analysis were done in parallel. In this way, the interviews incorporated new questions that emerged throughout the analysis. The interviewer tried to obtain detailed information by asking probing questions, rewording the same questions, and asking for examples or additional clarifications. The in-depth interviews had a duration of 40–100 min. They were audio recorded and transcribed literally. The audio files were destroyed afterwards.

### 2.3. Data Analysis

The content analysis of discourses was performed by a thematic analysis of the data [19] with the help of the computer program ATLAS.ti 8.0 (Scientific Software Development GmbH, Berlin, Germany). The informers’ accounts were first open-coded in large segments which were grouped thematically. The analysis was inductive, without previous categories that guided the coding. This was followed by axial coding to identify the relationships between the codes created. We analyzed each new data element collected, and we developed codes. When new codes appeared, we reviewed the data elements previously coded, and, when necessary, they were recoded until saturated. Finally, the codes were reduced to topics after an inductive process that involved PA, PM, and JAM, where codes and topics were discussed. Through this process, an image was generated to interpret the participants’ perceptions. For increased reliability of the analysis, all transcriptions were read and coded independently following the COREQ guidelines [20] which ensure the quality of the study.

### 2.4. Ethical Considerations

This study was approved by the Clinical Research Ethics Committee of the Integrated Health Management Area of Talavera de la Reina (Code 8/18). All participants gave informed consent. Confidentiality was maintained throughout the study, and only the research team has had access to the data obtained.

## 3. Results

According to the methodological principles of a phenomenological design and following the data analysis describe above, we identified the features or characteristics in common in the narratives of participants. As a result of this analysis, we identified three main topics related to the implementation of the Dependency Act in Extremadura and the impact of the RDL 20/2012: (1) the benefit it has brought to citizens, (2) the difficulties in its implementation, and (3) the negative effect that the policies adopted during the economic crisis has had on the dependency care system and on dependent persons themselves.

### 3.1. Benefits of the Dependency Act in Extremadura

The assessors state that the Dependency Act has brought considerable and relevant benefits, on the one hand, in the consideration of dependent persons as beneficiaries of a subjective, universal, and equitable right, establishing and granting legal status to those who need a third person to perform basic ADL. On the other hand, it has brought a political, legislative, and economic stimulus to make possible the creation of new resources of very different types for the care of dependent persons. As one of the informers explains:

*“in addition to what has been achieved at a broader level, such as the recognition of a subjective right and the protection that this entails, in our region, what has been achieved is, above all, to provide care in the territory, in the people’s homes, in their place of origin, their living, social care environment,… because some people are in a different type of care service, to bring resources closer, services that previously were provided by the will of x association or organization, rather subject to solidarity, not simply because the recognition of a right”*.

(I13)

The Act has made possible the expansion, management, and strengthening of dependency care resources. It has allowed stabilization and universalization of the entitlement to public benefits and services from the Autonomous Regions. In this way, a growing network of devices has been established, mainly residences and day centers, both in urban and rural areas, that may make it easier for citizens to receive care in their usual social and cultural environment without having to leave their place of socialization and origin.

Moreover, as the professionals interviewed point out, it has been a source of job creation both in the public sector and in private companies that manage resources, especially services that provide home care and residential care for a specific number of hours. These increased jobs linked to the social and health care sector and to dependency care have been primarily held by women, thus professionalizing and facilitating the inclusion in the labour market of a sector with high levels of unemployment.

### 3.2. Implementation Difficulties

Despite the substantial benefits of the Dependency Act, its implementation has faced important difficulties. According to the assessors, the expectations raised at the beginning of the Act’s development have generated a good deal of frustration:

*“I tell people that it is like a just-born child, you give them all your love and all of a sudden you stop feeding them”*.

(I1)

Likewise, the professionals interviewed for this study show their disagreement with the lack of coordination and cohesion between the different policies on care for persons in situations of dependency in this region (disability, elderly people, social services), as well as the excessive bureaucratization of the process that significantly slows down citizens from getting the benefits and services they require in due time to meet their needs and their family’s needs:

*“we cannot deny it comes late, through excessive bureaucracy, and that, moreover, it comes through an assessment that is not sensitive enough”*.

(I1)

The assessors also perceive that, while the DAS evaluates properly the situations of dependency derived from physical disabilities, it leaves out certain conditions. This lack of sensitivity is particularly striking when it comes to consider the situations experienced by people with mental illness:

*“I think that people suffering from mental restrictions, or limitations, or deficiencies are especially affected. The scale is not well designed there (…), people with mental illness are completely unprotected against the scale we use, whatever they say”*.

(I9)

Similarly, some professionals point out the difficulties caused by the fact that the scale does not take into account social and/or cultural aspects that may be affecting the individual’s performance. Thus, the assessment is made in isolation from the person’s context and life course.

Some therapists also claim that the scale handicaps those who use technical aids and those who make considerable efforts to be independent. It also hinders the transformative potential of the Dependency Act for people’s everyday lives:

*“all the tools designed to identify needs are not sensitive to many key aspects in people’s lives: their social context, their life course, any previous access or non-access to other resources. But on top of that, that tool is not capable of measuring the transformation that the Act itself or the resources made available to that citizen, the capacity it has to improve quality of life”*.

(I4)

They also argue that an assessment that determines access to the subjective right to dependency care, and also to a series of services and benefits, should be further protected and not so dependent on subjective aspects. In this regard, many professionals consider it necessary to supervise the assessments and to control their quality, as well as unify and compare assessment criteria.

In addition, the assessors point out the pressure from the administration to carry out a growing number of assessments that may reduce the waiting list of people awaiting examination with the scale. There is considerable complaint that quickness and quantity take priority over quality in the assessment:

*“when people ask for the Dependency Act, they do it with an aim in mind, with an idea, that sometimes is something that makes no sense, that other times comes with much concern, with great need, How can I walk away without telling them? You know? And, off you go home because you have to make the more the better. As there is only a handful of us, let’s do it very quickly. Besides, assessments are often really difficult to make”*.

(I1)

The interviewees also highlight the inequalities present within the region when it comes to access assessment because waiting times can vary considerably depending on the place of residence. The user’s residence also determines whether they can access certain services and/or benefits since the territorial distribution of resources has given significant priority to urban locations over rural areas—especially those poorly connected or less populated.

Regarding benefits, the assessors consider that benefits for care in the family have been distorted from the beginning because they were supposed to be exceptional even though, in many cases, they were able to dignify the end of the working life for many women in rural areas who have been (and still are) the primary caregivers:

*“…in a community where you have no resources, you have no businesses and you have no services… You have to give what you have. And what is it that you have? People at home. People at home caring for others. Women at home caring for their parents or their children or their siblings”*.

(I3)

As for the services deployed by the Dependency Act, there is significant discontent over the need to make people fit the existing resources, without even considering that the beneficiary’s needs could be better suited by other different resources:

*“The Act planned a study of each person, considering their health conditions, their functional capacity, their social and family situation, and provides an economic or other benefit (attendance, rehabilitation) specifically for you. But it has come to nothing. Well, are you dependent? Alright then. Choose between this, this, and this. No, not this because you do not meet the requirement. Between this and this. Because I cannot afford this. Ahhh. You get this. But this does not suit me. So there is nothing else. Come on. If you get worse ask for a review”*.

(I4)

Similarly, the development of the services linked to the Dependency Act has put a strain on two services (home care services and residential care) compared to other resources provided for in the Act which have had little implementation, such as night care centers or personal assistance. In a similar way, many assessors call for greater oversight of the work of the companies providing these services, demanding that this supervision should not only be of “environments” to check whether the service that these companies provide in “a delegate way “ is appropriate.

Finally, all the occupational therapists agree on the limited development of the promotion of personal autonomy, even though it was a priori one of the fundamental aspects of the Dependency Act, or the possibility of making changes to the allocated resources in order to eventually adapt them to the needs of the users and their families—in other words, according to the professionals interviewed, failing to consider the transformative power of the promotion of personal autonomy in the daily life of dependent persons.

*“I think that there should be much more focus on promotion of personal autonomy. For me, this is deficient. We see people with a Degree I and we know and report that they should review the file in the future because without rehabilitation and without promotion of personal autonomy they are going to get worse, unless they go to private resources to get it where people can privately pay for their services”*.

(I8)

### 3.3. Dependency in Times of Crisis: the Impact of the Royal Decree/Law 20/2012.

The occupational therapists participating in this study agree on the question of whether dependency has been considered a second-class right. Thus, the political decisions taken by the Spanish Government during the worst years of the economic crisis, especially the Royal Decree 20/2012, add up to the Dependency Act’s under-funding in recent years:

*“it was not a repeal, but it was, virtually, of a very important part… and what is taken away is very hard to get back. And there we are now (…), no-one could ever imagine that someone could be denied the right to go to a school or receive education for two years because, since we do not have a budget for it, you have to wait there”*.

(I13)

The impact of the government measures adopted to address the crisis has had significant consequences on the dependency care system, among others: cuts in benefits, increased times for care, and modification of the implementation schedule. RDL 20/2012 means, first a “delay” with respect to the initial schedule for the incorporation into the system of persons with a Degree I (moderate) dependency. This delay has left thousands of people without receiving any type of care until in 2015. However, the accumulation of delays was such that many people in certain areas of the region have not received the benefits that they were entitled to:

*“… when they removed Degree I and then until 2015, that was the worst thing. Because, of course, Degree I was not included until 2015 and they only included, and they are still negotiating Degree I from 2009 and 2010. Because those with Degree I, you negotiate them, but you leave them a review sheet because they are all worse off”*.

(I10)

In addition to these measures, the scale was changed in 2011, though it became effective in 2012, coinciding with the austerity policies mentioned above. Added to this are other decisions such as the decrease in economic benefits, the cut in home help hours, the elimination of the caregivers’ social security contributions, and the tightening of the conditions that the caregivers had to fulfil. This, according to the assessors, is generating clear discrimination:

*“… if you have economic resources you will be able to access certain services because you are going to pay for them yourself, otherwise… if in addition, certain services should help you from increasing this situation of dependence and to improve the quality of life,… at least you are going to stay the same”*.

(I5)

The Dependency Act has eventually been shaped as a gateway for resources for people who do not “fit” properly into the parameters of “dependency” set forth in the Act or into the possibilities of a scale that is not sensitive to the circumstances of certain people.

*“the dependency has been used to solve all the problems of the rest of the services, social services, mental health, disability, well, what a mess”*.

(I5)

In this regard, the assessors claim that they are pressured to adapt the degrees of dependency of the beneficiaries to the services, while, in addition, the tools are not adequate for the task of assigning dependent people to services, especially residential services, as one of the informers say:

*“… obviously the DAS I think it is not a tool designed to be an access filter to services. By no means. But it is true that it is in the interest of all autonomous regions that dependent people access it because of the way it is designed. Maybe the problem is the method of financing and that this does not condition the access to certain services”*.

(I13)

Finally, many therapists consider that, in the days when the crisis was most harmful, the benefits for care in the family have been a tool to meet the needs of low-income families.

## 4. Discussion

The Dependency Act is the best example of policies to address long-term care, which put forward the idea that this is a social problem requiring support from the State rather than considering it a particular and family situation. According to the results obtained in this work, the dependency assessors point out that the Dependency Act has had important and significant benefits for citizens in situations of dependency, as well as their families: it recognizes the subjective right to care (in contrast to previous paradigms more connected with assistentialism or solidarity), it has increased the number of care resources [21], and it has helped to create jobs in a sector traditionally occupied by women and markedly precarized [22]. In this respect, according to the Commission for the Analysis of the Situation of the Dependency System [5], there has been an increase of 162,539 new affiliations in the dependency-related sector in Spain since the adoption of the Dependency Act until December 2016. Thus, the expansion of care services for persons in situations of dependency is one of the positive aspects most valued by participants. This is similar to the results reported by Martínez-Buján [23], who stated that Extremadura is one of the regions where social services have been most strengthened. However, since the initial conditions were markedly worse than in other regions, Extremadura still needs a major boost to develop proximity services in a large territory with a wide geographical spread [24].

Despite its benefits, the implementation of an Act that was controversial from the beginning has faced important difficulties both in financing [25] and organization, as well as in some measures and decisions taken. The implementation had to coordinate the participation of state, regional, and local actors, together with private companies, all of which did not always share the same interests [26]. This development has caused important situations of inequality in waiting times for the dependency assessment by the professionals designated by the administration, for Individualized Care Plans, and for receiving benefits and/or services—between different Autonomous Regions [2,27] and within a region. All this is reflected in the interviews conducted with the assessors from the region of Extremadura.

On the other hand, despite the relevance of the DAS to recognize a right and to access a series of services and benefits [11], too little research has been done on its impact and on the corrective measures that should be taken to improve its sensitivity, its subjectivity, and to better assess the situation of dependency of certain groups, especially people with mental illnesses [28] or situations not caused by health conditions.

Despite the exceptional nature of the “Economic Benefit for Care in the Family” established in the White Paper on Dependency, it became the most widely used measure of the Dependency Act, even though the act gave priority to social services over economic benefits. As one of the study participants notes, when there were no other resources in Extremadura to provide appropriate care for dependent people, the only available resource was used: families [23,29]. The model set out in the Dependency Act still places too much weight on care in the family, especially on women [30,31], perpetuating roles and situations where care falls almost exclusively to women, especially in rural areas, and thus making their emancipation and self-determination difficult. However, it should be noted that, for many women, dependency care tasks can provide a sense of identity, a role valued by the community [32]. These aspects should be taken into greater account when considering actions of empowerment and personal development with respect to women caregivers.

Additionally, the participants agree on the differences in care services between urban and rural areas. Families in rural settings do not want to take the dependent person to a residential facility until they are not able to take it anymore. This would not happen if the model was best suited to the social and cultural reality in the region. The fabric of family and to a lesser extent neighbourhood becomes the main support, especially in rural areas.

As the participants point out, many resources set forth in the Dependency Act have not been implemented or only incipiently. This is particularly striking regarding measures of dependency prevention and of promotion of personal autonomy. The RDL 20/2012 has brought about a change (mainly due to penalties in regions that continue to rely on economic benefits for care in the family rather than other services and benefits) that according to Martínez-Buján [23] has caused “the consolidation of a strategy of family care privatization through domestic service”. This commodification, via economic aids to pay for private services when those services cannot be provided by the public system, has caused a significant increase of companies in the dependency sector, and therefore a significantly increased hiring of women [22,33]. But those companies are usually somehow beyond public administration’s control and supervision when it comes to providing services to dependent persons and their families.

As various studies show, the political measures taken to deal with the economic crisis have had an important impact on dependency care [10,34,35]: beneficiaries’ rights, care supply, and public spending for dependency care are all reduced. Thus, given the impoverished situations of many dependent persons and their families, recent research, such as some participants in this study, show how “the present socio-economic context makes the dependency economic benefit act as an integration minimum income that allows poor caregivers to cover their needs for basic subsistence” [36].

As regards the limitations of the study, it is worth noting that all participants come from the same Spanish region. The socio-demographic characteristics of this territory are very different from other regions in Spain. This means other territorial variables that may influence the perception of participants assessing dependency are not considered in this study.

However, this study highlights how the Dependency Act has established itself as a political tool which has generated important social and economic benefits. It also reveals some of the main difficulties of its implementation, which may be common among different territories and may be resolved with sectorialised political actions.

## 5. Conclusions

According to the participants in charge of the assessment of situations of dependency in Extremadura, the implementation of the Dependency Act has had important benefits for dependent persons and their families. However, its development has faced a number of difficulties: lack of sensitivity of the scale of assessment, difficulties in the coordination of policies, under-funding, or the limited development of measures of promotion of personal autonomy.

Future research should also include the perspectives of the persons in situations of dependency and their families regarding the benefits of the Dependency Act. Likewise, it would be important to carry out research that allows determining whether there are differences between the experiences regarding the assessment of women participants and men participants. It would also be important to consider the influence of the participants’ area of origin on their experience when analysing assessments.

## Figures and Tables

**Table 1:** Gender and type of population in which participants work.

Participant	Gender	Type of Population
I1	Woman	Rural
I2	Woman	Rural
I3	Man	Rural
I4	Woman	Rural
I5	Woman	Rural/Urban
I6	Woman	Rural/Urban
I7	Man	Urban
I8	Woman	Rural
I9	Man	Rural/Urban
I10	Woman	Rural
I11	Woman	Rural/Urban
I12	Woman	Urban
I13	Man	Urban
I14	Woman	Rural/Urban
I15	Man	Rural
I16	Woman	Rural

**Table 2:** Semi-structured interview script.

● How do you rate the implementation of the Dependency Act in Extremadura?
● In your opinion, what aspects can be improved in the implementation of the Dependency Act in Extremadura?
● What is the situation of dependent people and their families in the territory where you make assessments?
● What are the future challenges for the care of dependent persons and their families?
● How does the DAS assess dependency in different health and/or disability conditions? *

* This last question did not appear in the initial script and was added after analysing the first interviews.
